# Supplementary material for: Phenotypic and Functional Alterations of Immune Effectors in Periodontitis; A Multifactorial and Complex Oral Disease
Source: J Clin Med. 2021 Feb 20;10(4):875. doi: 10.3390/jcm10040875 (PMC7924323; doi:10.3390/jcm10040875)
Supplement: Supplementary file 1 [file jcm-10-00875-s001.pdf]

Supplementary file

Figure S1: Increased NK cells in oral blood in comparison to peripheral blood in periodontitis patients

Mononuclear cells were isolated from peripheral and oral blood of periodontitis patients using Ficoll-Hypaque density gradient. The percentages of CD16+CD56+ NK cells were determined immediately after purification using specific antibody staining followed by flow cytometric analysis. IgG2 isotype antibodies were used as controls. One of four representative experiments is shown in figure.

Figure S2: Characteristics of lymphocytes obtained from gingival tissues

Mononuclear cells were purified from gingival tissues of periodontitis patients as described in the Materials and Methods section and the surface expression levels of CD45 and CD69 were determined using staining with specific antibodies followed by flow cytometric analysis. IgG2 isotype antibodies were used as controls **(A)**. The surface expression levels of CD45, CD28, and CD95 were determined using staining with specific antibodies followed by flow cytometric analysis. IgG2 isotype antibodies were used as controls **(B)**. One of four representative experiments is shown in figure.

Figure S3: Increased activation of NK and CD8+ T cells in oral blood as compared to peripheral blood of periodontitis patients

CD16+ and CD8+ cells (1×10^6/ml) obtained from oral blood and peripheral blood of periodontitis patients were treated with a combination of PMA (10 ng/ml) and Ionomycin (10 ng/ml) for 18-24 hours after which they were washed twice. The surface expression levels of CD16 and CD69 for NK cells, and CD8 and CD69 for T cells were determined using staining with specific antibodies followed by flow cytometric analysis. IgG2 isotype antibodies were used as controls. Numbers in each quadrant represent

the percentages of stained sub-population for the specific antigen. One of four representative experiments is shown in this figure.

Figure S4: Increased cytotoxicity, IFN-γ secretion, and decreased IL-6 in co-culture of NK cells with NFkB knockdown OSCCs and HOK-16B cells

IκB(S32AS36A) transduced OSCCs **(A)**, and IκB(S32AS36A) transfected HOK-16B **(B)** and their EGFP transduced controls were transfected with 8 μg of NFkB Luciferase reporter vector and treated with and without TNF-*α* (20 ng/ml) for 18 hours. The relative Luciferase activity was then determined in the lysates and fold induction in the luciferase activity was calculated relative to untreated cells **(A, B)**. NK cells were left untreated and treated with IL-2 (1000 U/ml) for 18-20 hours before they were added to ^{51}Cr-labeled IκB(S32AS36A) transduced OSCCs **(C)**, and IκB(S32AS36A) transfected HOK-16B **(D)** and their EGFP transduced controls at various effector-to-target ratios. NK cell-mediated cytotoxicity was measured using a standard 4-hour ^{51}Cr release assay. The lytic units (LU) 30/10^6 cells were determined using the inverse number of NK cells required to lyse 30% of target cells × 100 **(C, D)**. NK cells were left untreated and treated with IL-2 (1000 U/ml) for 18-20 hours before they were co-cultured with IκB(S32AS36A) transduced OSCCs **(E)**, and IκB(S32AS36A) transfected HOK-16B **(F)** and their EGFP transduced controls at 1:1 ratio. After overnight incubation, the supernatants were harvested to determine IFN-γ secretion using single ELISA **(E, F)**. IκB(S32AS36A) transduced OSCCs **(G)**, and IκB(S32AS36A) transfected HOK-16B **(H)** and their EGFP transduced controls were cultured at 2 × 10^5 cells/ml, after overnight incubation, the supernatants were harvested to determine IL-6 secretion using single ELISA **(G, H)**. One of four representative experiments is shown in this figure. ***(*p* value <0.001), **(*p* value 0.001-0.01), *(*p* value 0.01-0.05).

Figure S5: Cytokine secretion levels in *F. nucleatum* treated PBMCs co-cultured with HEp2-pRcCMV, and HEp2-IκB(S32AS36A) cells

PBMCs were left untreated and treated with IL-2 (500 U/ml) in the presence or absence of *F. nucleatum* overnight before they were co-cultured with HEp2-pRcCMV, and HEp2-IκB(S32AS36A) cells (bacteria: PBMCs: Hep2 tumors at 30:2:1 ratios). After overnight incubation, the supernatants were harvested to determine cytokine secretion levels were determined using multiplex assay. One of four representative experiments is shown in this figure. ***(*p* value <0.001), **(*p* value 0.001-0.01), *(*p* value 0.01-0.05).

Figure S6: Determined cytokine secretion levels in *F. nucleatum* treated NK cells co-cultured with HEp2-pRcCMV, and HEp2-IκB(S32AS36A) cells

NK cells were left untreated and treated with IL-2 (500 U/ml) in the presence or absence of *F. nucleatum* overnight before they were co-cultured with HEp2-pRcCMV, and HEp2-IκB(S32AS36A) cells (bacteria: PBMCs: Hep2 tumors at 30:2:1 ratios). After overnight incubation, the supernatants were harvested to determine cytokine secretion levels were determined using multiplex assay. One of four representative experiments is shown in this figure. ***(*p* value <0.001), **(*p* value 0.001-0.01), *(*p* value 0.01-0.05).

Figure S1

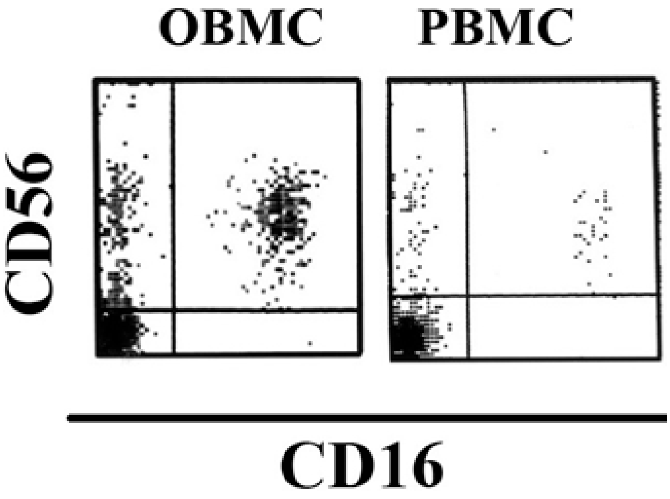

Figure S2

A B

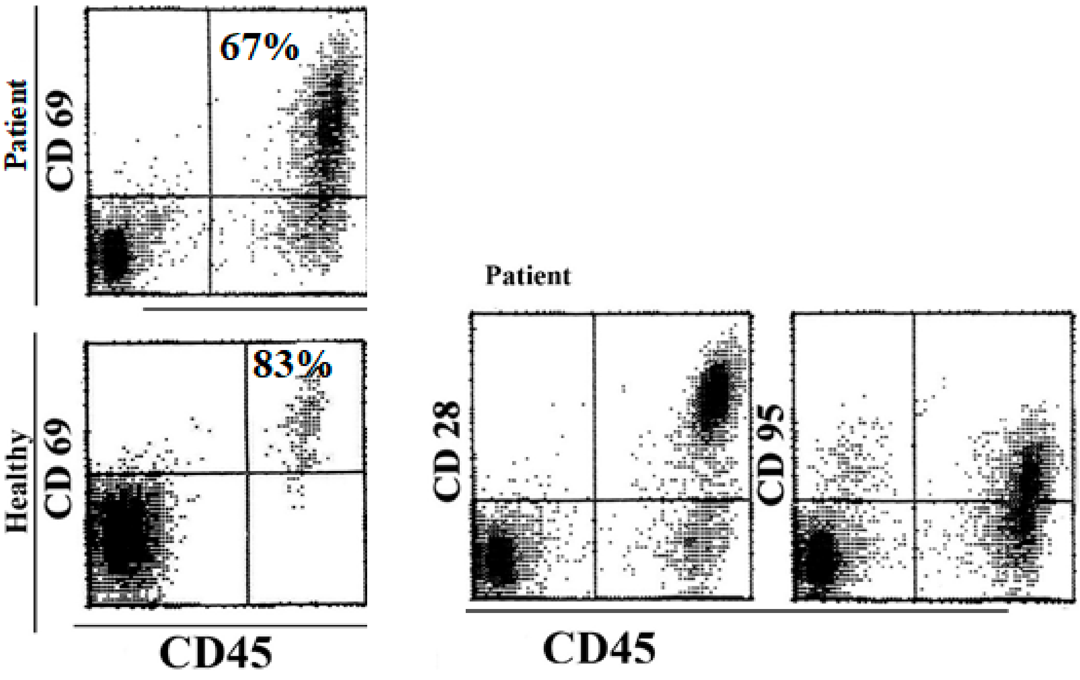

Figure S3

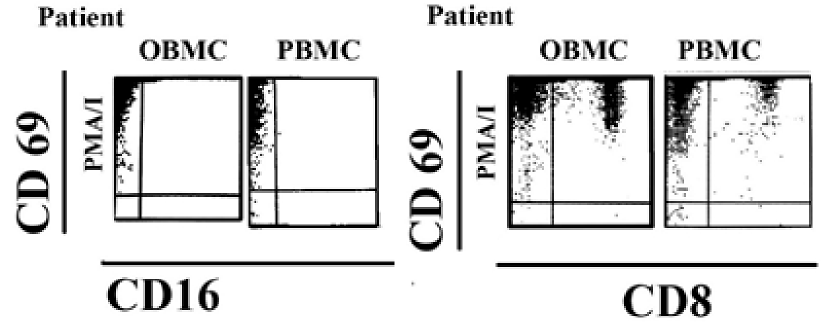

Patient
OBMC
PBMC
CD 69
PMA/I
CD16
Patient
OBMC
PBMC
CD 69
PMA/I
CD8

Figure S4

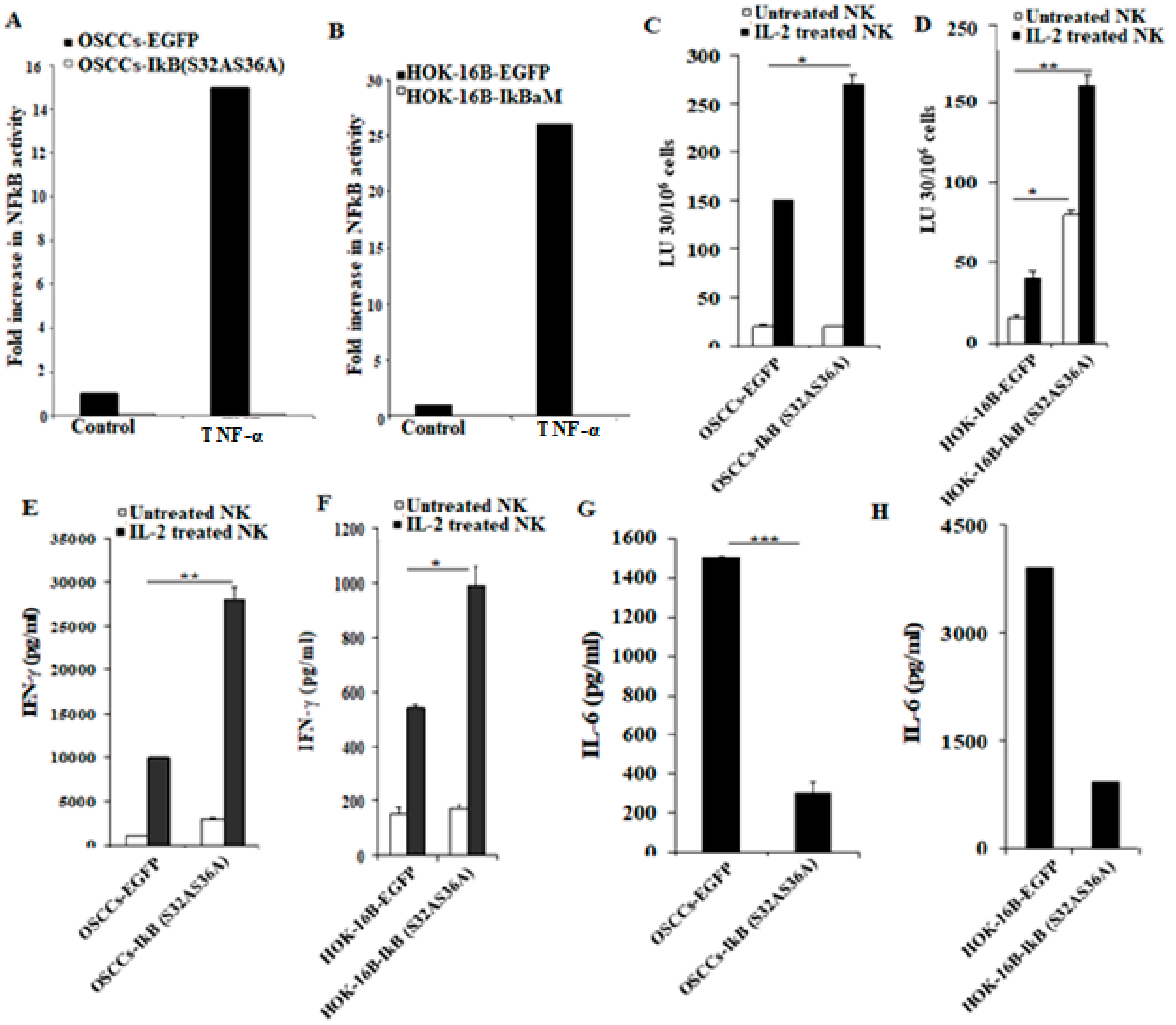
A
OSCCs-EGFP
OSCCs-IkB(S32AS36A)
Fold increase in NFkB activity
Control
TNF-α
B
HOK-16B-EGFP
HOK-16B-IkBaM
Fold increase in NFkB activity
Control
TNF-α
C
Untreated NK
IL-2 treated NK
LU 30/10⁶ cells
OSCCs-EGFP
OSCCs-IkB (S32AS36A)
D
Untreated NK
IL-2 treated NK
LU 30/10⁶ cells
HOK-16B-EGFP
HOK-16B-IkB (S32AS36A)
E
Untreated NK
IL-2 treated NK
IFN-γ (pg/ml)
OSCCs-EGFP
OSCCs-IkB (S32AS36A)
F
Untreated NK
IL-2 treated NK
IFN-γ (pg/ml)
HOK-16B-EGFP
HOK-16B-IkB (S32AS36A)
G
IL-6 (pg/ml)
OSCCs-EGFP
OSCCs-IkB (S32AS36A)
H
IL-6 (pg/ml)
HOK-16B-EGFP
HOK-16B-IkB (S32AS36A)

Figure S5

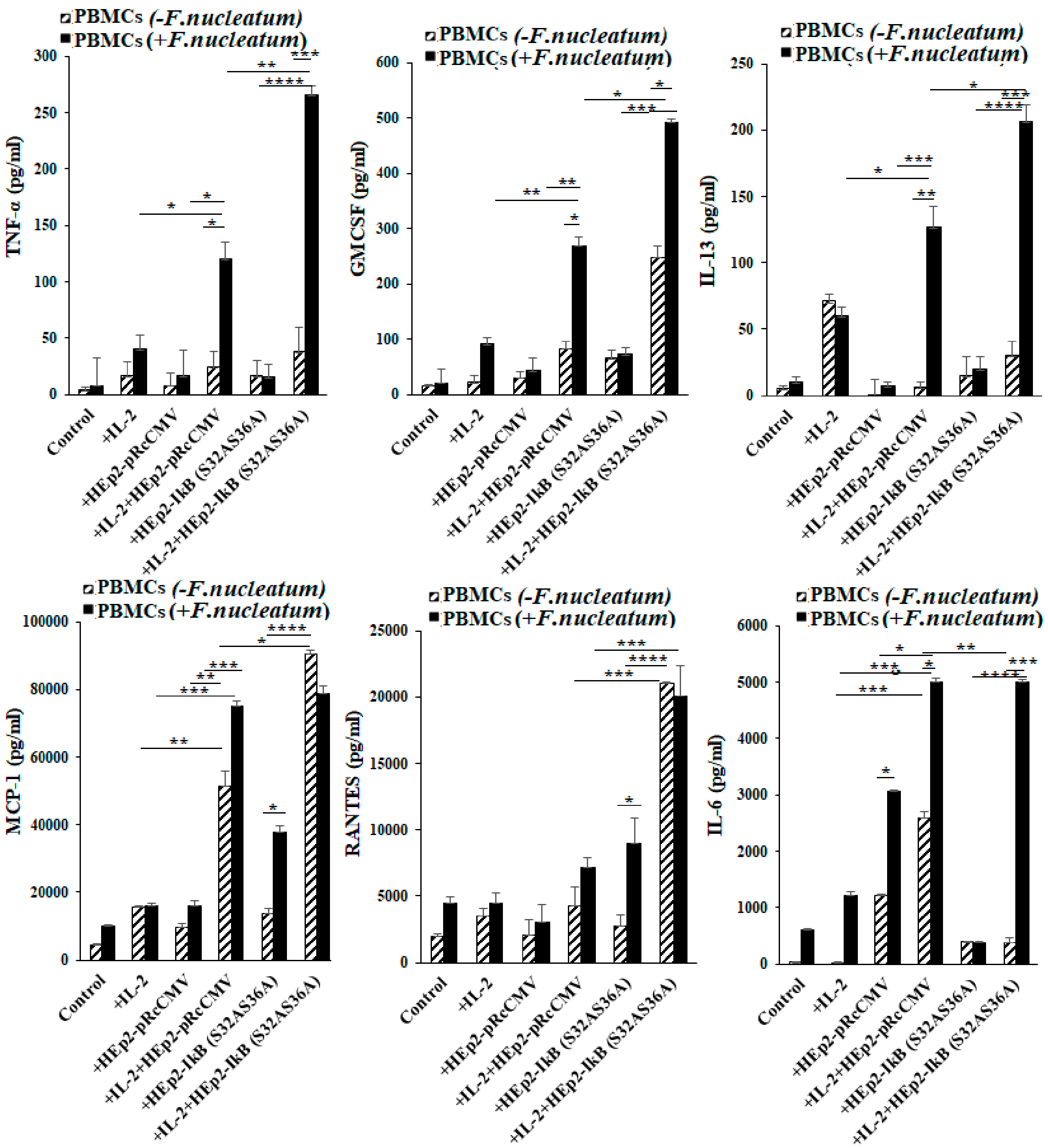
PBMCs (-F.nucleatum)
PBMCs (+F.nucleatum)
TNF-α (pg/ml)
GMCSF (pg/ml)
IL-13 (pg/ml)
MCP-1 (pg/ml)
RANTES (pg/ml)
IL-6 (pg/ml)
Control
+IL-2
+HEp2-pRcCMV
+IL-2+HEp2-pRcCMV
+HEp2-IkB (S32AS36A)
+IL-2+HEp2-IkB (S32AS36A)

Figure S6

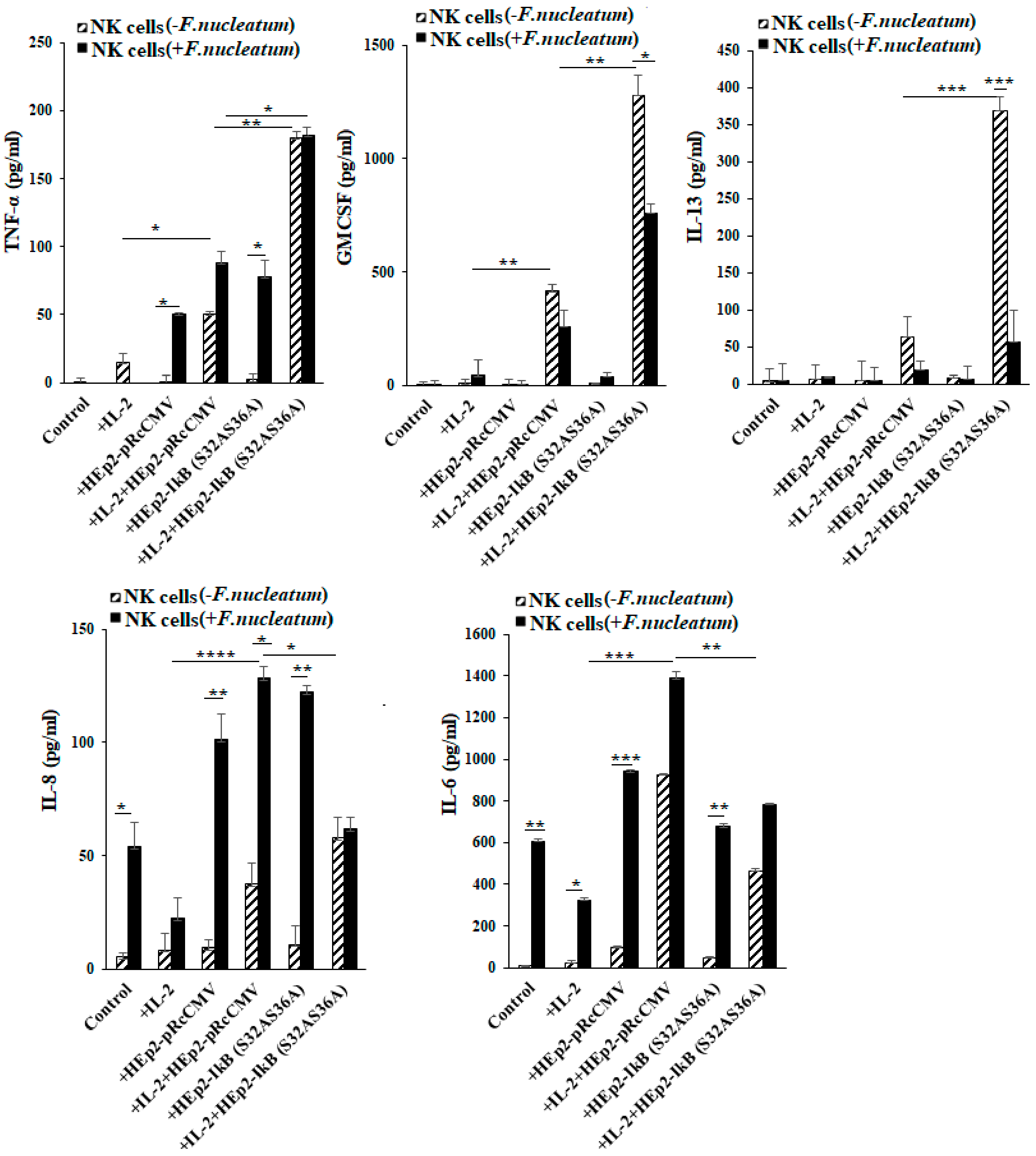

NK cells(-F.nucleatum)
NK cells(+F.nucleatum)
TNF-α (pg/ml)
GMCSF (pg/ml)
IL-13 (pg/ml)
IL-8 (pg/ml)
IL-6 (pg/ml)
Control
+IL-2
+HEp2-pRcCMV
+IL-2+HEp2-pRcCMV
+HEp2-IkB (S32AS36A)
+IL-2+HEp2-IkB (S32AS36A)
